# Differential diagnosis of common etiologies of left ventricular hypertrophy using a hybrid CNN-LSTM model

**DOI:** 10.1038/s41598-022-25467-w

**Published:** 2022-12-05

**Authors:** In-Chang Hwang, Dongjun Choi, You-Jung Choi, Lia Ju, Myeongju Kim, Ji-Eun Hong, Hyun-Jung Lee, Yeonyee E. Yoon, Jun-Bean Park, Seung-Pyo Lee, Hyung-Kwan Kim, Yong-Jin Kim, Goo-Yeong Cho

**Affiliations:** 1 Cardiovascular Center, Seoul National University Bundang Hospital, 82 Gumi-Ro-173-Gil, Bundang, Seongnam, Gyeonggi 13620 South Korea; 2 Department of Internal Medicine, Seoul National University College of Medicine, Seoul, South Korea; 3 Center for Artificial Intelligence in Healthcare, Seoul National University Bundang Hospital, Songnam, Gyeonggi South Korea; 4 Division of Cardiology, Cardiovascular Center, Korea University Guro Hospital, Seoul, South Korea; 5 Cardiovascular Center and Department of Internal Medicine, Seoul National University Hospital, Seoul, South Korea

**Keywords:** Cardiology, Medical research

## Abstract

Differential diagnosis of left ventricular hypertrophy (LVH) is often obscure on echocardiography and requires numerous additional tests. We aimed to develop a deep learning algorithm to aid in the differentiation of common etiologies of LVH (i.e. hypertensive heart disease [HHD], hypertrophic cardiomyopathy [HCM], and light-chain cardiac amyloidosis [ALCA]) on echocardiographic images. Echocardiograms in 5 standard views (parasternal long-axis, parasternal short-axis, apical 4-chamber, apical 2-chamber, and apical 3-chamber) were obtained from 930 subjects: 112 with HHD, 191 with HCM, 81 with ALCA and 546 normal subjects. The study population was divided into training (n = 620), validation (n = 155), and test sets (n = 155). A convolutional neural network-long short-term memory (CNN-LSTM) algorithm was constructed to independently classify the 3 diagnoses on each view, and the final diagnosis was made by an aggregate network based on the simultaneously predicted probabilities of HCM, HCM, and ALCA. Diagnostic performance of the algorithm was evaluated by the area under the receiver operating characteristic curve (AUC), and accuracy was evaluated by the confusion matrix. The deep learning algorithm was trained and verified using the training and validation sets, respectively. In the test set, the average AUC across the five standard views was 0.962, 0.982 and 0.996 for HHD, HCM and CA, respectively. The overall diagnostic accuracy was significantly higher for the deep learning algorithm (92.3%) than for echocardiography specialists (80.0% and 80.6%). In the present study, we developed a deep learning algorithm for the differential diagnosis of 3 common LVH etiologies (HHD, HCM and ALCA) by applying a hybrid CNN-LSTM model and aggregate network to standard echocardiographic images. The high diagnostic performance of our deep learning algorithm suggests that the use of deep learning can improve the diagnostic process in patients with LVH.

## Introduction

Echocardiography is widely accepted as an essential diagnostic tool for cardiovascular evaluation. Most measurements on echocardiography can be automated using machine learning techniques^1–3^. However, the value of echocardiography also includes differential diagnosis and clinical decision making. Echocardiography specialists make judgements based on the visual information from echocardiographic images, along with knowledge and experience. Because of complex and diverse medical situations, the interpretation of echocardiographic images and resulting decision still remain as dependent on the clinician’s expertise.

The differential diagnosis of “unexplained” left ventricular hypertrophy (LVH) on echocardiography is important, but challenging^4^. LVH is most commonly a physiologic consequence of increased afterload by hypertension (i.e. hypertensive heart disease [HHD])^5^. However, some patients demonstrate hypertrophied myocardium without an increased afterload; the differential diagnosis in such patients includes hypertrophic cardiomyopathy (HCM) and infiltrative cardiomyopathy, such as light-chain cardiac amyloidosis (ALCA)^4,6–9^. The differential diagnosis of LVH requires a series of expensive, invasive, and time-consuming procedures, such as cardiac magnetic resonance imaging (CMR), endomyocardial biopsy (EMB), and genetic testing^4^. In particular, CMR is useful in the differentiation of LVH of unknown etiology based on the well-established typical CMR features of HCM and ALCA, but is expensive and sometimes unavailable, and does not confirm the diagnosis^10,11^. For confirmation of the diagnosis, EMB is useful, especially for ALCA. However, EMB has limitations such as invasiveness, lower diagnostic yield at the right ventricle (RV), difficulty in approaching to the LV myocardium, and a lack of specific histologic markers for HHD^12,13^. Genetic testing can be useful for the detection of HCM, but the results are often inconclusive and sometimes do not provide confirmative diagnostic information^14^. Due to these limitations, patients with LVH of unknown etiology require additional tests, which necessitate substantial time and cost. More importantly, these tests often need to be performed simultaneously or sequentially, as the findings of each test might not provide confirmative results. If the echocardiographic findings can narrow the differential diagnosis of LVH of unknown etiology, then the time and cost required for diagnostic process can be reduced, and patients can avoid unnecessary tests. However, although echocardiography plays a role in screening for the suggestion of differential diagnosis of “unexplained” LVH, this imaging modality might not be correct, and may mislead or complicate the diagnostic process^15^. Therefore, the presumptive diagnosis by expert cardiologists must be improved in terms of accuracy, and there is a clinical need for higher diagnostic accuracy on echocardiography for a more efficient diagnostic process.

Considering that machine learning can objectively evaluate imaging data without prejudice, and construct a decision from information that is difficult for human eyes to comprehend, it can be assumed that a machine learning approach would be helpful for the differential diagnosis of LVH on echocardiography. Therefore, in the present study, we aimed to differentiate common LVH etiologies (HHD, HCM, and ALCA) on standard echocardiographic images by using a hybrid convolutional neural network-long short-term memory (CNN-LSTM) algorithm.

## Methods

**Figure 1:**
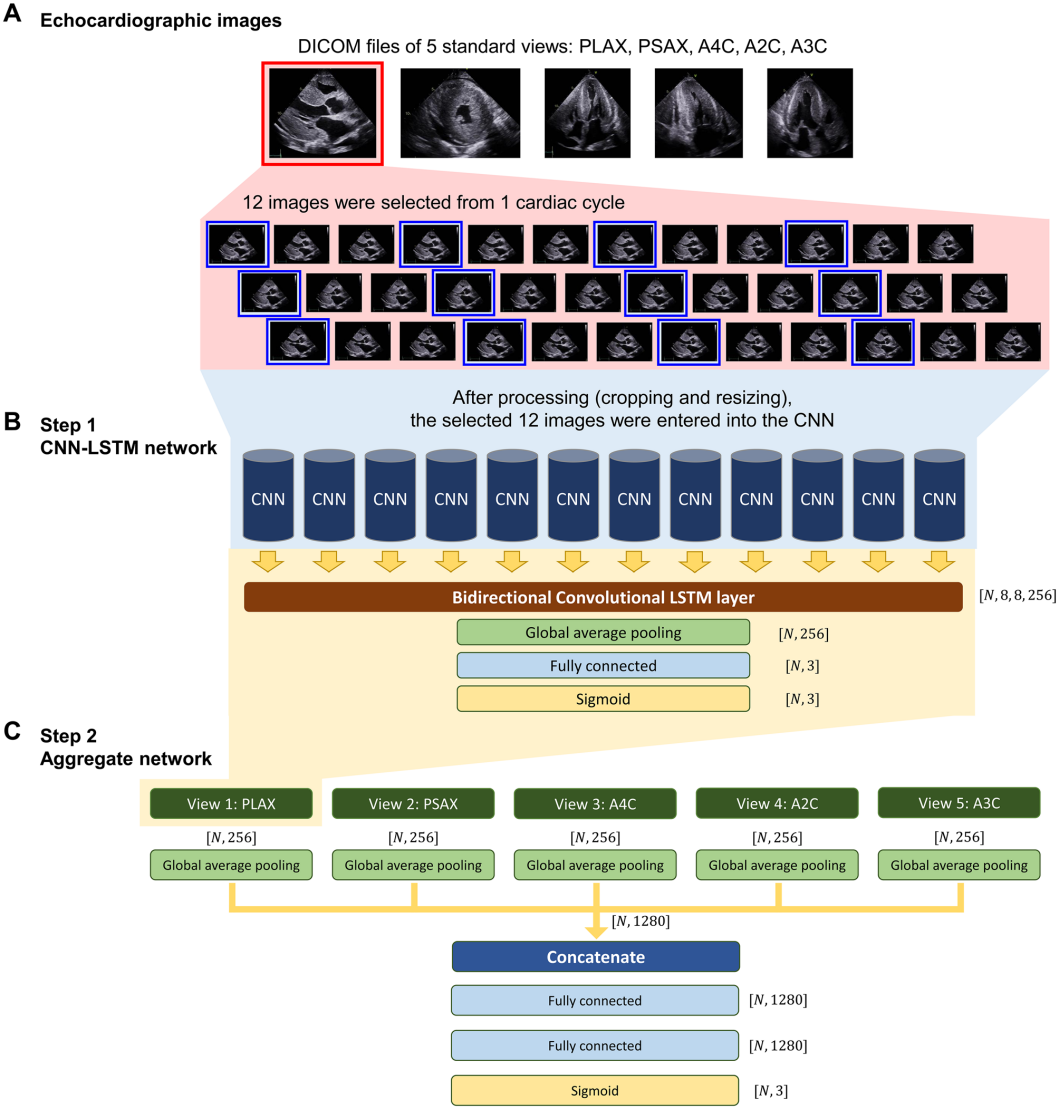
Development of the CNN-LSTM model and aggregate network. Schematic figure depicting the development of the deep learning algorithm. (**A**) Twelve DICOM images were extracted from 1 cardiac cycle at the same time-interval, for each of the 5 standard echocardiographic views. (**B**) In the first step of model development, a CNN-LSTM network was developed for each of the 5 standard echocardiographic views. The same CNN was applied to the 12 echocardiographic images, and a bi-directional convolutional LSTM layer was then applied to these 12 CNNs. A CNN-LSTM network was produced for each of the 5 echocardiographic views. (**C**) In the second step, an aggregate neural network was developed using the outputs obtained from the global average pooling of the multi-label classification block in each of 5 independent CNN-LSTM networks. *A2C* apical 2-chamber view, *A3C* apical 3-chamber view, *A4C* apical 4-chamber view, *CNN* convolutional neural network, *DICOM* digital imaging and communications in medicine, *LSTM* long short-term memory, *PLAX* parasternal long-axis view, *PSAX* parasternal short-axis view.

The overall scheme of the study is depicted in Fig. 1 and more detailed methods are available in the Supplementary Methods.

### Study design and cohort

This study conformed to the principles outlined in the Declaration of Helsinki and was approved by the Seoul National University Bundang Hospital Institutional Review Board (IRB No. B-2105-687-107) in May 2021. The requirement for informed consent was waived by the Seoul National University Bundang Hospital Institutional Review Board because of the retrospective nature of the study and minimal expected risk to the subjects. This study was conducted and described according to the Proposed Requirements for Cardiovascular Imaging-Related Machine Learning Evaluation, as suggested by the American College of Cardiology Healthcare Innovation Council^16^.

From the echocardiography databases of Seoul National University Bundang Hospital (n = 755) and Seoul National University Hospital (n = 175), we retrospectively identified 930 subjects (112 patients with HHD, 191 with HCM, 81 with ALCA, and 546 normal subjects). The diagnostic criteria for HHD, HCM and ALCA are described below.

#### HHD

Patients with a history of hypertension, who met the diagnostic criteria for LVH on echocardiography (LV mass index [LVMI] > 115 g/m^2^ in men, and > 95 g/m^2^ in women) were included^17,18^. The following additional criteria were required for a specific diagnosis of HHD: (1) end-diastolic maximal LV wall thickness (LVWTmax) ≥ 12 mm, (2) regression of LVH after appropriate blood pressure control, and (3) exclusion of other causes of LVH (such as HCM, infiltrative cardiomyopathy, metabolic cardiomyopathy, etc.).

#### HCM

Patients who met the diagnostic criteria of HCM (LVWTmax ≥ 15 mm on echocardiography, in the absence of abnormal loading conditions that could sufficiently explain the LVH) were included^19,20^. For a specific and accurate diagnosis of HCM, definite evidence of HCM on CMR or a typical gene mutation on genetic analysis were required.

#### ALCA

According to clinical guidelines, ALCA on echocardiography was suggested when the LVWTmax was > 12 mm^21^. Other typical features on echocardiography, such as (1) symmetrical LV thickening; (2) right ventricular (RV) free wall thickening; (3) small pericardial effusion; (4) thickening of the atrioventricular valves and interatrial septum; (5) abnormal myocardial texture characterized as a speckled appearance; (6) voltage-mass discrepancy; (7) base-to-apex strain gradient or relative apical sparing of longitudinal strain; and (8) typical findings on CMR (patchy, subendocardial circumferential, or diffuse fuzzy late gadolinium enhancement [LGE] of the LV), were used for clinical suspicion and detection of ALCA^21,22^. For a specific and accurate labeling, definite evidence of amyloid involvement on EMB was required. Due to the small number of patients with transthyretin amyloidosis and potential differences in myocardial texture, we included patients with ALCA, and excluded those with transthyretin amyloidosis.

#### Normal subjects

Inclusion criteria for normal subjects were as follows: (1) no clinical history of cardiovascular disease or diabetes; (2) normal blood pressure (≤ 130/80 mm Hg); (3) body mass index ≤ 30 kg/m^2^; (4) normal sinus rhythm at 50–85 beats/min without conduction abnormalities; (5) normal LV wall thickness, LV wall motion, and left atrial volume (< 27 mL/m^2^ using the biplane method of discs); (6) no mitral valve prolapse; and (7) no more than trivial valve regurgitation.

#### Exclusion criteria

Patients were excluded if they had (1) significant LV dysfunction (LV ejection fraction < 40%), (2) active malignancy (or receiving chemotherapy), (3) end-stage renal disease, (4) prior coronary revascularization, (5) significant valve disease, (6) regional wall motion abnormality, (7) no evidence of LVH or LVWTmax < 11 mm, or (8) other metabolic or infiltrative cardiomyopathies, such as Fabry disease, Danon disease, mitochondrial encephalopathy lactic acidosis and stroke-like episodes (MELAS), and PRKAG2 cardiomyopathy.

### Echocardiography

All images were obtained using a standard ultrasound device with a 2.5-MHz probe, in accordance with the guidelines of the American Society of Echocardiography^17^. Echocardiograms comprised 1 cardiac cycle, obtained in 5 standard views (parasternal long-axis [PLAX], parasternal short-axis [PSAX], apical 4-chamber [A4C], apical 2-chamber [A2C], and apical 3-chamber [A3C]).

### Image processing for the deep learning algorithm

Echocardiogram videos were downloaded as Digital Imaging and Communications in Medicine (DICOM) files from the picture archiving and communication system, and anonymized (Fig. 1). Because of differences in heart rate and echocardiographic frame rate, the number of images in the cardiac cycle differed among patients and views. Therefore, 12 images were extracted at the same interval for each view. The extracted images were cropped to 12 × 12cm^2^ based on each center point to remove parts not related to the region of interest. The cropped images were resized to 256 × 256 pixels using bilinear interpolation. Pydicom (python package, version 2.1.0) was used to preprocess the DICOM files.

### Deep learning model development

The development of the deep learning model is detailed in the Supplementary Methods. Briefly, the total study population (n = 930) was divided into training (n = 620), validation (n = 155), and test sets (n = 155). Using the training set, a deep learning algorithm based on a CNN-LSTM for the differential diagnosis of LVH was developed in two major steps (Fig. 1). The first step comprised the development of a CNN-LSTM network^23,24^. The same CNN was applied to the 12 DICOM images extracted from each standard echocardiographic view. Because we aimed to combine the CNN’s feature extraction from the DICOM images and the LSTM’s temporal information, we opted to extract 12 images/cardiac cycle, in order to avoid exhaustive amount of computing time from various lengths of input videos, while maintaining clinical relevance^25^. Then, in order to reflect the temporal and spatial connectivity between the 12 DICOM images, a bi-directional convolutional LSTM layer was applied. Finally, a multi-label classification block was applied to predict HHD, HCM, and ALCA independently, on each view. The second step comprised the development of a neural network that aggregated the results obtained in the first step. This neural network was developed to decide the final “most-likely” diagnosis among 4 categories (normal, HHD, HCM, and ALCA) from the 5 standard views of each patient; in real-world clinical practice, the evaluation of a patient’s echocardiographic images should lead to a single clinical diagnosis. The outputs obtained from the 5 independent CNN-LSTM networks were concatenated to compose the input. Binary cross entropy was used as an objective function to train the first and second steps, and *He*-initialization was used to initialize the weights^26^. The region to which the deep learning algorithm reacted sensitively in images was detected using class activation mapping^27^. Network development was implemented using the Tensorflow framework (version 2.3) and graphic processing unit (NVIDIA GeForce RTX 2080 Ti) in Linux (Ubuntu 16.04) with NVIDIA CUDA/cuDNN (versions 10.1 and 7.6, respectively).

### Study outcomes

The study outcomes were the area under the receiver operating characteristic curve (AUC) for the differentiation of the 4 categories (normal, HHD, HCM, and ALCA) and the diagnostic accuracy as calculated by the confusion matrix. For the latter, the final diagnosis made by the deep learning algorithm was compared to the ground-truth labeling. Additionally, using the test set, the diagnostic performance of the CNN-LSTM model was evaluated by comparing the final diagnosis made by the deep learning algorithm to the visual interpretation of expert cardiologists (I-C Hwang and G-Y Cho, who have more than 10 and 25 years of experience in echocardiography, respectively).

### Statistical analysis

The AUC was used to measure the classification performance. Sensitivity, specificity, positive and negative predictive values, and positive and negative likelihood ratios of the deep learning algorithm were calculated for each disease. The optimal cutoff for each of the 3 diseases was calculated in advance using the Youden’s J statistic of the validation set^28^. If the probabilities for HHD, HCM, or ALCA were smaller than the corresponding optimal cutoff, the diagnosis was “normal”. Otherwise, the highest value among the probabilities for HHD, HCM, and ALCA decided the final diagnosis. Cohen’s $\kappa$ coefficient and the confusion matrix were calculated to compare the diagnostic performance between the deep learning algorithm and the expert clinicians^29^. Diagnostic accuracy based on the confusion matrix was calculated as (true positives + true negatives)/(true positives + true negatives + false positives + false negatives). All statistical analyses were performed using R statistical software version 4.1.1 (The R Foundation for Statistical Computing, Vienna, Austria). p-values < 0.05 were considered statistically significant.

## Results

### Baseline characteristics

In total, 4650 echocardiograms from 930 subjects (5 standard echocardiographic views for each subject) were analyzed. Baseline characteristics of the study population are summarized in Table 1. The LVWTmax and LVMI were significantly larger in patients with LVH than in normal subjects, but there were no significant differences between HHD, HCM, and CA subgroups. Details regarding the composition of the training, validation, and test sets are provided in Table 2.

**Table 1:** Baseline characteristics.

	Normal subjects (n = 546)	HHD (n = 112)	HCM (n = 191)	ALCA (n = 81)	*p*-value
Age (years)	48.2 ± 12.0	49.5 ± 14.8	55.3 ± 13.3	68.3 ± 11.2	< 0.001
Male sex	260 (47.6%)	77 (68.8%)	121 (63.4%)	43 (53.1%)	< 0.001
Height (cm)	165.4 ± 9.0	167.8 ± 9.1	164.4 ± 10.0	160.1 ± 9.0	< 0.001
Weight (kg)	65.6 ± 11.9	75.9 ± 17.2	69.0 ± 12.3	57.5 ± 11.1	< 0.001
BMI (kg/m^2^)	23.9 ± 3.1	26.4 ± 5.3	25.4 ± 3.5	21.9 ± 4.5	< 0.001
Systolic blood pressure (mmHg)	124.2 ± 14.3	159.7 ± 26.4	127.6 ± 17.9	113.3 ± 18.8	< 0.001
Diastolic blood pressure (mmHg)	74.8 ± 9.2	96.5 ± 19.8	75.0 ± 11.1	69.0 ± 13.4	< 0.001
**Echocardiographic parameters**					
LV-EDD (mm)	46.2 ± 3.8	49.7 ± 5.2	45.0 ± 5.2	43.5 ± 5.4	< 0.001
LV-ESD (mm)	29.5 ± 3.7	34.1 ± 7.2	26.7 ± 4.5	29.8 ± 5.7	< 0.001
LV-EDV (ml)	81.1 ± 19.3	105.7 ± 35.3	76.1 ± 20.8	64.7 ± 18.0	< 0.001
LV-ESV (ml)	29.6 ± 8.0	46.5 ± 23.1	26.4 ± 8.0	27.9 ± 11.8	< 0.001
LV-EF (%)	63.6 ± 4.1	57.6 ± 9.2	64.9 ± 6.7	57.5 ± 8.8	< 0.001
IVSd (mm)	8.8 ± 1.2	13.8 ± 2.5	17.2 ± 4.8	13.8 ± 2.5	< 0.001
LVPWd (mm)	8.5 ± 1.1	13.0 ± 2.2	11.6 ± 2.9	13.2 ± 2.3	< 0.001
LV-MI (gm/m^2^)	75.8 ± 13.7	146.8 ± 36.0	150.3 ± 46.6	138.1 ± 34.6	< 0.001
LA dimension (mm)	34.4 ± 4.3	40.6 ± 6.5	43.7 ± 6.6	41.7 ± 6.5	< 0.001
LAVI (mL/m^2^)	29.0 ± 5.5	40.8 ± 14.0	48.3 ± 19.4	55.8 ± 19.0	< 0.001
Mitral E/e’ ratio	7.7 ± 1.9	13.0 ± 4.7	16.3 ± 8.0	23.7 ± 10.7	< 0.001
TR Vmax (m/sec)	2.1 ± 0.3	2.3 ± 0.6	2.4 ± 0.5	2.6 ± 0.6	< 0.001
PASP (mmHg)	24.5 ± 4.4	29.4 ± 10.8	32.2 ± 7.8	36.4 ± 10.6	< 0.001

Values are given as the median with interquartile range or as a number (percentage).

*AF* atrial fibrillation, *BMI* body-mass index, *ALCA* light-chain cardiac amyloidosis, *EDD* end-diastolic dimension, *EDV* end-diastolic volume, *EF* ejection fraction, *ESD* end-systolic dimension, *ESV* end-systolic volume, *HHD* hypertensive heart disease, *HCM* hypertrophic cardiomyopathy, *IVSd* interventricular septum at end-diastole, *LVPWd* left ventricular posterior wall thickness at end-diastole, *LVWTmax* end-diastolic maximal LV wall thickness, *LA* left atrium, *LAVI* left atrial volume index, *LV* left ventricular, *MI* mass index, *N/A* not applicable, *PASP* pulmonary artery systolic pressure, *TR* tricuspid regurgitation.

**Table 2:** Splitting of the data into training, validation and test sets.

Groups	Data split	Normal	HHD	HCM	ALCA
1	Training set	91	19	32	13
2		91	19	32	13
3		91	18	32	14
4		91	19	32	13
5	Validation set	91	19	31	14
6	Test set	91	18	32	14
Total (n = 930)		545	112	191	81

Total study population was split into 6 groups, of which 4 groups were designated as the training set, and remaining 2 groups designated as the validation and test sets, respectively.

*AF* atrial fibrillation, *BMI* body-mass index, *ALCA* light-chain cardiac amyloidosis, *EDD* end-diastolic dimension, *EDV* end-diastolic volume, *EF* ejection fraction, *ESD* end-systolic dimension, *ESV* end-systolic volume, *HHD* hypertensive heart disease, *HCM* hypertrophic cardiomyopathy, *IVSd* interventricular septum at end-diastole, *LVPWd* left ventricular posterior wall thickness at end-diastole, *LVWTmax* end-diastolic maximal LV wall thickness, *LA* left atrium, *LAVI* left atrial volume index, *LV* left ventricular, *MI* mass index, *N/A* not applicable, *PASP* pulmonary artery systolic pressure, *TR* tricuspid regurgitation.

### Diagnostic accuracy

The diagnostic accuracy of the developed algorithm was assessed at each step of the algorithm development. First, the AUCs for the differential diagnosis of HHD, HCM, and ALCA were obtained from the CNN models without LSTM network, and were compared with the AUCs obtained from the CNN-LSTM model (Supplementary Table S1). In overall, the AUCs for the diagnosis of HHD, HCM and ALCA were higher with the combined CNN-LSTM model compared to the CNN models: the averaged AUCs of the CNN models were around 0.9, but further improved by the addition of LSTM network. Details regarding the diagnostic performance for each view are provided in Supplementary Table S2, comparing the diagnosis made by the expert cardiologists and that by CNN-LSTM algorithm.

**Figure 2:**
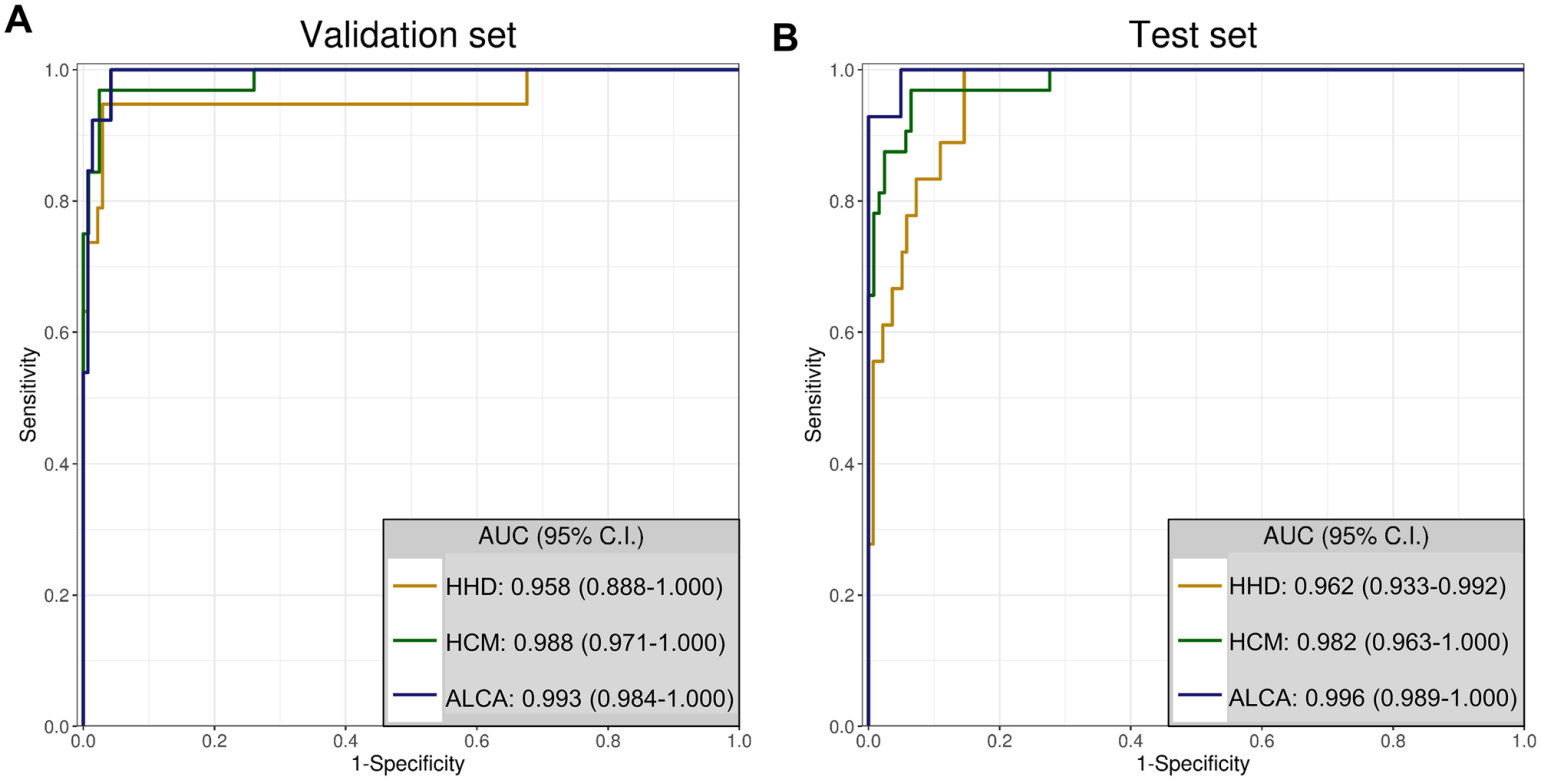
ROC curve analysis of the deep learning algorithm for the differential diagnosis of LVH. The diagnostic accuracy of the deep learning algorithm was calculated using the AUC for the validation (**A**), and test sets (**B**). This figure was generated using R software v.4.1.1, R Core Team (2021). R: A language and environment for statistical computing. R Foundation for Statistical Computing, Vienna, Austria. URL http://www.r-project.org/. *AUC* area under the ROC curve, *CI* confidence interval, *ALCA* light-chain cardiac amyloidosis, *HCM* hypertrophic cardiomyopathy, *HHD* hypertensive heart disease, *ROC* receiver operating characteristic curve.

Second, the AUCs obtained from the combined CNN-LSTM model of each echocardiographic views and those of the final AUCs from the final aggregate network of the 5 standard views were assessed in the validation and test sets (Table 3, Supplementary Fig. S1). In the validation set, the AUCs of the final aggregate network of the 5 standard echocardiographic views were 0.958, 0.988, and 0.993, for the diagnosis of HHD, HCM, and ALCA, respectively (Table 3, Fig. 2A). The AUCs were similar in the test set (0.962, 0.982 and 0.996, respectively) (Table 3, Fig. 2B). The AUCs obtained from the final aggregate network were higher than those from each echocardiographic view. Details on the sensitivity, specificity, positive predictive value (PPV), and negative predictive value (NPV) for each LVH etiology, provided by the expert cardiologists and CNN-LSTM algorithm, are compared in Supplementary Table S3.

**Table 3:** AUCs for the differential diagnosis of LVH.

Echocardiographic view	Validation set			Test set		
	AUC for HHD	AUC for HCM	AUC for ALCA	AUC for HHD	AUC for HCM	AUC for ALCA
PLAX	0.954	0.974	0.976	0.949	0.968	0.951
PSAX	0.894	0.97	0.871	0.892	0.959	0.929
A4C	0.931	0.954	0.953	0.908	0.973	0.989
A2C	0.905	0.925	0.916	0.917	0.974	0.978
A3C	0.908	0.983	0.924	0.907	0.937	0.986
Aggregate	0.958	0.988	0.993	0.962	0.982	0.996

The AUC for each differential diagnosis (HHD, HCM and CA) was calculated in the validation (n = 155) and test sets (n = 155).

*A2C* apical 2-chamber view, *A3C* apical 3-chamber view, *A4C* apical 4-chamber view, *PLAX* parasternal long-axis view, *PSAX* parasternal short-axis view, *AF* atrial fibrillation, *BMI* body-mass index, *ALCA* light-chain cardiac amyloidosis, *EDD* end-diastolic dimension, *EDV* end-diastolic volume, *EF* ejection fraction, *ESD* end-systolic dimension, *ESV* end-systolic volume, *HHD* hypertensive heart disease, *HCM* hypertrophic cardiomyopathy, *IVSd* interventricular septum at end-diastole, *LVPWd* left ventricular posterior wall thickness at end-diastole, *LVWTmax* end-diastolic maximal LV wall thickness, *LA* left atrium, *LAVI* left atrial volume index, *LV* left ventricular, *MI* mass index, *N/A* not applicable, *PASP* pulmonary artery systolic pressure, *TR* tricuspid regurgitation.

The diagnostic performance of the CNN-LSTM model and aggregate network was compared according to the number of echocardiographic images extracted from 1 cardiac cycle, which was one of the major hyperparameters of our deep learning algorithm (Supplementary Methods and Supplementary Table S4). In the developed model, the number of images/cardiac cycle was determined empirically: 12 DICOM images were extracted from 1 cardiac cycle, considering the various heart rates and frame rates of the included echocardiogram videos. The AUCs of the algorithm based on 12 images/cardiac cycle were comparable to the models based on 4, 8, or 16 images/cardiac cycle. In order to reflect the entire cardiac cycle in echocardiogram videos of various heart rates and frame rates in routine clinical practice, the 12 images/cardiac cycle was maintained for the deep learning algorithm. Further, the AUCs were compared between the 2-dimensional (2D) image-based CNN-LSTM model with aggregate network and the 3-dimensional CNN (3D-CNN) model, which was suggested in a recent study^30^. In this analysis, we extracted 12 images/cardiac cycle or 16 images/cardiac cycle for the 3D-CNN model for comparability with our 2D-CNN-LSTM model, and found that the AUCs of the 3D-CNN model are not significantly different compared to our algorithm (Supplementary Table S5).

### Echocardiographic features used in the differential diagnosis

**Figure 3:**
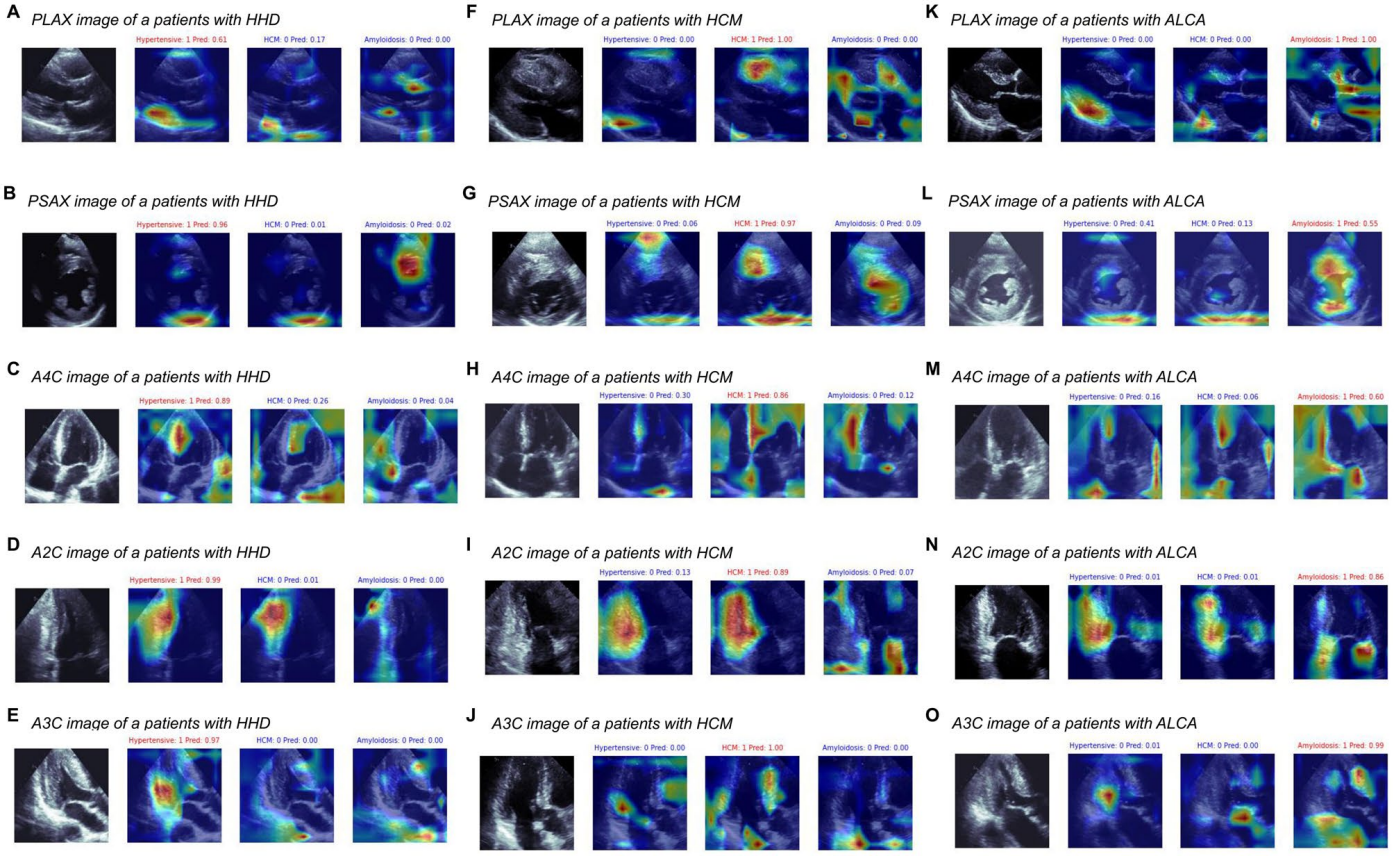
Representative figures in the class activation mapping. Typical class activation maps of patients with HHD (**A–E**), HCM (**F–J**), or ALCA (**K–O**), are presented. *AUC* area under the ROC curve, *CI* confidence interval, *ALCA* light-chain cardiac amyloidosis, *HCM* hypertrophic cardiomyopathy, *HHD* hypertensive heart disease, *ROC* receiver operating characteristic curve.

Class activation mapping demonstrated that well-established typical echocardiographic findings for the differential diagnosis of LVH were utilized in the deep learning algorithm (Fig. 3 and Supplementary Table S6). In PLAX views, the highlighted regions comprised the anteroseptum, ascending aorta, and basal inferolateral segment with posterior mitral valve leaflet (Fig. 3A,F,K). In PSAX views, the septum and papillary muscle were highlighted in all 4 categories, and for the differentiation of ALCA, the pericardium at the LV posterior side was highlighted (Fig. 3B,G,L). The inferoseptum and papillary muscle were typically highlighted in A4C images (Fig. 3C,H,M); the LV inferior wall and LA wall were highlighted in A2C images (Fig. 3D,I,N); and the anteroseptum, inferolateral wall, and the pericardium at the LV posterior side were highlighted in A3C images (Fig. 3E,J,O). The frequencies of the highlighted regions in each echocardiographic view are summarized in Supplementary Table S6.

### Comparison with expert interpretation

**Figure 4:**
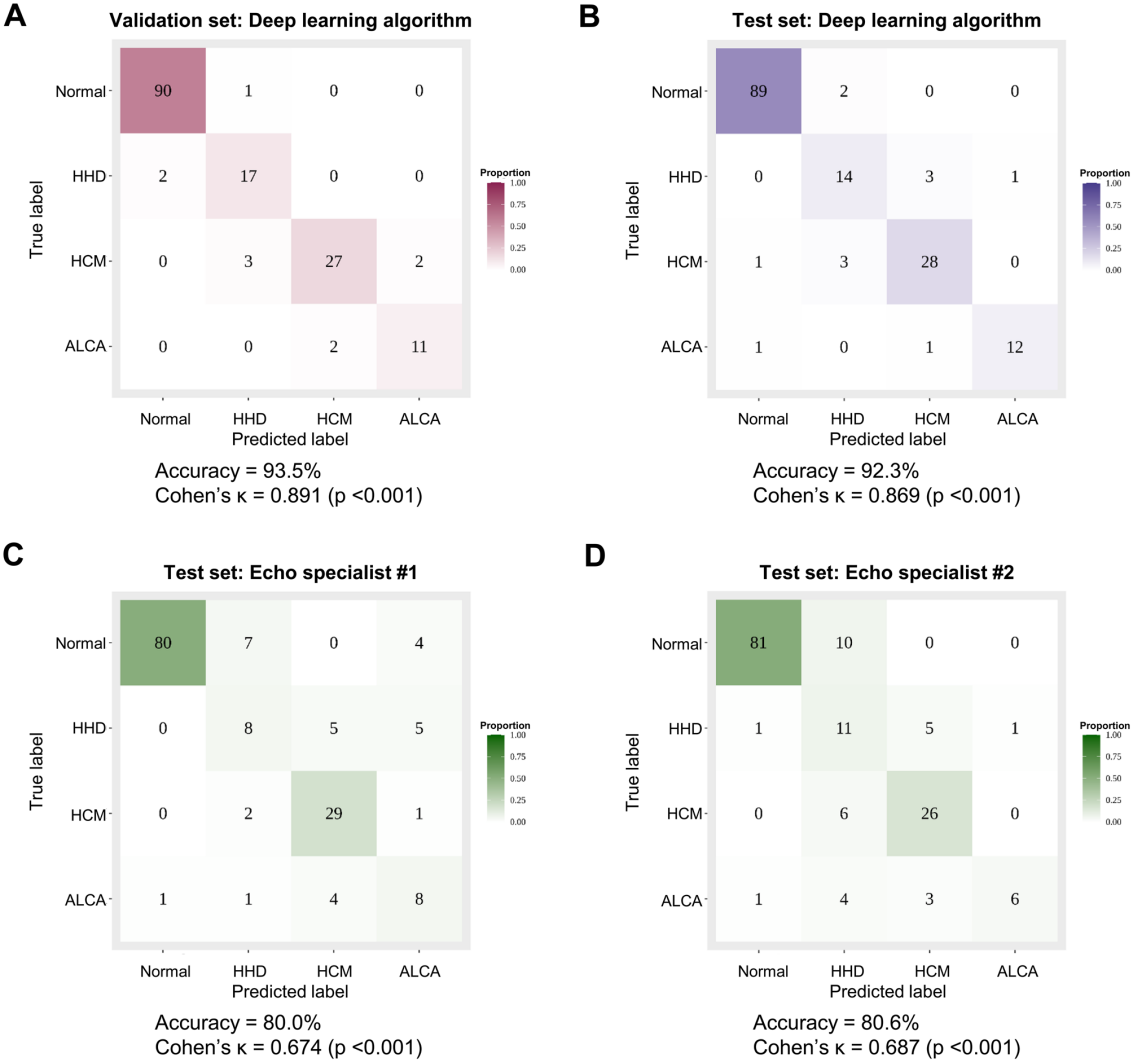
Diagnostic accuracy of the deep learning algorithm compared to that of expert cardiologists. The diagnostic accuracy of the deep learning algorithm was assessed using the confusion matrix for the validation (**A**), and test sets (**B**). The accuracy was also compared to that of two echocardiography specialists using the test set (**C**,**D**). This figure was generated using R software v.4.1.1, R Core Team (2021). R: A language and environment for statistical computing. R Foundation for Statistical Computing, Vienna, Austria. URL http://www.r-project.org/. *AUC* area under the ROC curve, *CI* confidence interval, *ALCA* light-chain cardiac amyloidosis, *HCM* hypertrophic cardiomyopathy, *HHD* hypertensive heart disease, *ROC* receiver operating characteristic curve.

As shown in Supplementary Table S2, the diagnostic performance of expert cardiologists on a single echocardiographic view was not satisfactory: the sensitivity ranged from 14 to 78% and the PPV from 26 to 77%. Although the diagnostic performance of expert cardiologists was improved when the 5 standard echocardiographic views were combined for decision, the sensitivity, specificity, PPV and NPV for each LVH etiology were lower than those provided by the deep learning algorithm using the hybrid CNN-LSTM model and aggregate network (Supplementary Table S3). The overall diagnostic accuracy of the deep learning algorithm was 92.3% and the Cohen’s $\kappa$ was 0.869 (p < 0.001), which were significantly higher than those of the two expert cardiologists (expert 1: accuracy, 80%; Cohen’s $\kappa$, 0.674; p < 0.001; expert 2: accuracy, 80.6%; Cohen’s $\kappa$, 0.687; p < 0.001) (Fig. 4).

## Discussion

In the present study, we developed a deep learning algorithm based on 5 standard echocardiographic views from 930 subjects to differentiate the common etiologies of LVH on echocardiography using a hybrid CNN-LSTM model and aggregate network. The deep learning algorithm showed excellent diagnostic performance in the differentiation of LVH, which was significantly greater than that based on expert cardiologists’ interpretations of the echocardiogram. These findings suggest that deep learning-assisted interpretation of the echocardiogram can improve the accuracy of the differential diagnosis of LVH, and improve the overall diagnostic process.

### Etiologies of LVH and challenges for differential diagnosis

LVH is often a physiologic adaptation to an increased afterload, with a prevalence reaching 10% to 15% in the echocardiography laboratory^31^. However, the etiology of LVH is not limited to hypertension, but includes a wide range of disease conditions. According to previous echocardiographic studies, the common causes of LVH other than HHD include HCM and CA^9^. HCM is a genetic disease with an approximate prevalence of 1:200–1:500, and the patients with HCM show significant LVH due to myocardial fiber disorganization/disarray^19,20^. Light-chain CA is a hematologic malignant disease, in which abnormally increased amyloid protein production leads to a profound infiltration of amyloid protein in the myocardium, resulting in significant LVH^32^.

The differential diagnosis between these conditions is important because of differences in the treatment and prognosis. While the management of HHD mainly focuses on blood pressure control, the management of HCM and ALCA is much more complex and multifactorial. In patients with HCM, the treatment strategy includes sudden cardiac death risk assessment; primary or secondary prevention of sudden death; management of combined arrhythmia, heart failure, or LV outflow tract obstruction; and family counseling/screening^19,20^. The management of ALCA includes cytotoxic chemotherapy and stem cell transplantation, along with the management of cardiovascular complications such as arrhythmia and heart failure^21^. Furthermore, the overall life expectancy of patients with HCM is comparable to that of the general population, but 30–40% of patients will experience adverse events^19^. In contrast, patients with light-chain ALCA have a very poor prognosis, with a median survival from the initial diagnosis of only 24 months^22,32^.

Although the underlying LVH pathophysiology differs between HHD (increased afterload), HCM (sarcomere mutation and myofibril disarray/disorganization), and ALCA (amyloid protein infiltration), the differential diagnosis is often difficult on echocardiography. This is because of morphologic similarities on echocardiography, and the high prevalence of hypertension in patients with HCM or ALCA^6,7,15^. The differential diagnosis of HCM is especially problematic when patients show diffuse or mixed-type HCM. A comprehensive echocardiography examination can improve the diagnostic accuracy in the detection of ALCA, which paradoxically suggests that the visual assessment has limited use in the differential diagnosis^8,33,34^. The difficulties in the differential diagnosis on echocardiography leads to the subsequent use of numerous noninvasive and invasive tests, such as CMR, EMB, and genetic testing. However, despite the limited diagnostic accuracy in many clinical situations, these tests often require additional cost, time, and invasiveness^10–14^. Thus, improvements in the differential diagnosis of LVH etiologies by echocardiography can facilitate the efficient diagnostic process, and further lead to a timely application of disease-specific treatment.

### Relevance of an artificial intelligence-supported differential diagnosis

Our deep learning algorithm showed excellent diagnostic accuracy for the differential diagnosis of LVH using 5 standard echocardiographic views. It might be argued that the differences in the LV wall thickness might be the determinant of the differential diagnosis, given that the patients with ALCA may have less LVH than HHD or HCM. However, in the present study, the inclusion criterion of LVWTmax was > 12 mm for both HHD and ALCA, and the mean LV wall thickness did not differ between the two groups. Due to innate characteristics of the deep learning process, as well as relatively small study population, we cannot provide detailed reasons for this improvement or delicate sensitivity analyses; however, the class activation mapping results provided clues. For the diagnosis of HHD, the class activation map highlighted regions at the ascending aorta on PLAX views, RV insertion site on PSAX views, and RV apex and LV inferior/inferolateral wall on apical views (A4C, A2C and A3C) (Fig. 3 and Supplementary Table S6). Patients with HHD show concentric or eccentric LVH, but specific echocardiographic findings differentiating HHD from other causes of LVH are largely unknown. However, a previous CMR study reported that patients with HHD demonstrate LGE at RV insertion points, and limited aortic distensibility, which might have been utilized in our deep learning algorithm^35^. The diagnosis of HCM was mainly based on highlighted regions at the basal septum and basal inferolateral wall on PLAX views, and the LV septum and inferior wall on apical views, all of which are typically hypertrophied in patients with HCM^20,36^. For the diagnosis of ALCA, highlighted regions typically included the anterior mitral valve leaflet, left atrial wall, and LV basal inferior/inferolateral segments with the adjacent pericardial space. Patients with ALCA often demonstrate thickened valve leaflets and atrial wall due to amyloid protein infiltration, and a small amount of pericardial effusion^37^. Although not sufficiently pathognomonic to exclude other possible differential diagnoses, these highlighted regions show typical features for the clinical suspicion and determination of LVH etiologies on echocardiography.

Furthermore, it can be assumed that different myocardial textures and motions were also utilized in the deep learning algorithm, as suggested in a recent study by *Fei Yu *et al.^38^. In particular, the microscopic features of LVH etiologies significantly differ, due to different underlying pathophysiology. Patients with HHD have hypertrophied cardiomyocytes with diffuse myocardial fibrosis, whereas patients with HCM typically have disorganized myocardial fibers with marked fibrosis, and those with ALCA have infiltrated amyloid proteins. These pathophysiologic differences are also utilized in the visual assessment of echocardiographic images (e.g. increased echogenicity in HCM, and a granular sparkling appearance in ALCA). However, visual interpretation of these morphologic features is subjective to the observer’s discretion, and thus, is not specific. In the current class activation mapping results, a thickened LV myocardium was highlighted in most echocardiographic images, suggesting that the myocardial texture was utilized as an important indicator in the differential diagnosis.

In the present study, it was noted that the PPV values of the CNN-LSTM algorithms for each standard echocardiographic view were low, ranging from 30 to 70% (Supplementary Table S2). Thus, we applied the aggregate network in order to concatenate the results obtained from the CNN-LSTM models of 5 echocardiographic views. The concatenated outputs from the aggregate network significantly improved the overall diagnostic performance, as well as the PPV values. The use of aggregate network resembles in part the clinical decision by human experts, in which a full series of echocardiographic images are integrated. Given that the highlighted regions on class activation mapping differed between the 5 echocardiographic views, it can be inferred that the aggregate network could improve diagnostic accuracy through integration of features from the 5 different views. In addition, the diagnostic accuracy of our deep learning algorithm was significantly higher than that for the echocardiography specialists. In real-world practice, the overall diagnostic process for unexplained LVH is guided by the decisions of echocardiography specialists. Thus, the higher diagnostic accuracy, especially the excellent NPV and specificity, of our deep learning algorithm can contribute to a more efficient process, reducing the time and effort required for a final diagnosis of the LVH etiology. Although a deep learning algorithm-assisted diagnosis on echocardiography cannot yet replace the current confirmative diagnostic tools, this approach can help attending physicians go straight to confirmative testing, avoiding inconclusive results and uncertain debates regarding the diagnosis.

### Machine-learning approaches for differential diagnosis of LVH etiologies

The application of deep learning in echocardiography has been considered as challenging, because of the various view orientations and inter-view differences as well as the variability within a single view^39^. However, several landmark studies demonstrated accurate view classification with segmentation, cardiac structure identification, and cardiac phase detection, all of which enabled the accurate automated measurement of cardiac structures and functional parameters^2,3,40–42^. These can contribute to the accurate measurement of echocardiographic parameters while reducing human errors. On top of these, the deep learning algorithms demonstrated promising results in the detection of certain echocardiographic features, such as the presence of LVH or regional wall motion abnormalities^39,43^, and furthermore, differential diagnosis on echocardiographic images to aid clinical decision-making, which was previously believed to require complex and sophisticated clinical reasoning by specialists. In particular, several studies focused on the differential diagnosis of LVH and demonstrated meaningful results.

A study by *Xiang Yu *et al*.* also developed a deep learning algorithm for detection of LVH and its differential diagnosis of HHD, HCM, and ALCA^44^, but we found that the methodology is different compared to our study. The study by *Xiang Yu *et al*.* obtained 2 still images from PLAX and A4C views of each patient, utilized the ResNet and *U*-net ++ for the algorithm development, and performed manual delineation of LV myocardium as the ground truth. In contrast, we obtained 5 standard echocardiogram videos (PLAX, PSAX, A4C, A2C, and A3C) and extracted 12 images from 1 cardiac cycle, in order to reflect the motion of cardiac structures. Our deep learning algorithm did not require the manual delineation of cardiac structures, but provided excellent diagnostic accuracy and demonstrated that relevant echocardiographic features were utilized for the decision, as shown in the class activation map. In addition, we tried our best effort to improve the diagnostic accuracy of our deep learning algorithm, avoiding the use of images from repetitive echocardiograms from a same patient, which is another difference compared to the study by *Xiang Yu *et al.^44^. Furthermore, we confirmed that each step of the algorithm development, such as the application of LSTM network and the use of aggregate network, improved the diagnostic accuracy. Indeed, the combined CNN-LSTM algorithm was adopted to appropriately reflect the myocardial texture, along with myocardial systolic and diastolic motions. The LSTM algorithm is a novel and efficient type of recurrent neural network, and has strengths in time series prediction, such as in movie frames. Because the myocardial systolic and diastolic motions can differ between HHD, HCM, and ALCA, these features might have been utilized in our deep learning algorithm.

More recently, *Duffy *et al. developed a deep learning workflow for measurement of LV geometry and diagnosis of LVH etiologies, using a large-scale cohort of 23,745 patients^30^. In that landmark study, a deep learning model for measurement of LV dimensions and wall thickness was developed using PLAX videos, and a video-based CNN model for identification of the etiology was developed using A4C videos. One of the important differences of the study by *Duffy *et al. compared to our study is the use of 3D-CNN with spatiotemporal convolutions. In contrast, we designed 2D-based CNN for 12 images extracted from 1 cardiac cycle in order to extract echocardiographic features for differential diagnosis. Then, an LSTM layer was applied to the 12 CNNs, to reflect the temporal and special changes of the heart during the cardiac cycle. Given the relevance of both methods (2D-CNN-LSTM and 3D-CNN) for acquisition of spatiotemporal data, we compared the diagnostic performance of these methods using our dataset (Supplementary Table S5), and found that the AUCs were not different. These findings infer that, echocardiographic features including geometry, myocardial texture, and cardiac systolic/diastolic motions, can be reflected in both 2D-CNN-LSTM algorithm and 3D-CNN algorithm. Another important difference is the echocardiographic views used in the study. The deep learning algorithm developed by *Xiang Yu *et al. utilized 2 still images of echocardiogram (PLAX and A4C)^44^, and the algorithm by *Duffy *et al. utilized only A4C videos for the differential diagnosis of LVH^30^. In contrast, we utilized 5 standard echocardiographic views (PLAX, PSAX, A4C, A2C and A3C) for the development of CNN-LSTM algorithms, which were concatenated to provide a single most-likely diagnosis. Although it can be assumed that the integration of various aspects of cardiac structure and function may improve the diagnostic accuracy, future studies on the direct comparison of these algorithms are required. Furthermore, given the potential benefits of a deep learning-assisted differential diagnosis, prospective studies or clinical trials are warranted to assess whether its use can reduce the time, costs, and number of tests deemed as necessary, compared to that for echocardiography specialists alone.

## Limitations

The present study has several limitations. First, we did not include rare LVH etiologies, such as Fabry disease, MELAS, Danon syndrome, PRKAG2 cardiomyopathy, and transthyretin amyloidosis. The exclusion of these rare diseases was inevitable to ensure a sufficient number of patients for each LVH etiology. However, future multi-center studies are warranted to include the rare LVH etiologies in the deep learning algorithm. Second, we excluded patients with valvular heart disease or chronic kidney disease, as there is a possibility that these conditions overlap with the LVH etiologies included in the present study. Nonetheless, the overlap of these conditions cannot be strictly classified into a specific label, and it is impossible to clearly distinguish the proportion of each causative factor of LVH. Third, we excluded patients with other overt echocardiographic abnormalities, such as regional wall motion abnormalities or significant LV dysfunction. As the presence of these pathologic conditions indicate a poor prognosis in patients with LVH, future studies are warranted to develop a comprehensive deep learning algorithm that includes a wide range of complex cardiac conditions. Finally, our deep learning algorithm was developed using echocardiographic images from 2 tertiary hospitals in South Korea, but was not validated in external datasets from other ethnicities. For further validation, as well as for facilitation of deep learning approaches in cardiovascular imaging, the full code for our algorithm was released (https://github.com/djchoi1742/Echo_LVH).

## Conclusion

We developed a deep learning algorithm for the differential diagnosis of common LVH etiologies (HHD, HCM, and ALCA) by applying a hybrid CNN-LSTM model and aggregate network to standard echocardiographic images. The high diagnostic performance of our deep learning algorithm suggests that the use of deep learning can improve the diagnostic process in patients with LVH.

## Supplementary Information

Supplementary Information.

## Data Availability

The datasets of echocardiographic images generated during and/or analysed during the current study are available from the corresponding author on reasonable request. The full code for our algorithm was released (https://github.com/djchoi1742/Echo_LVH).
