# Supplementary material for: Differential diagnosis of common etiologies of left ventricular hypertrophy using a hybrid CNN-LSTM model
Source: Sci Rep. 2022 Dec 5;12:20998. doi: 10.1038/s41598-022-25467-w (PMC9722705; doi:10.1038/s41598-022-25467-w)
Supplement: Supplementary file 1 — Supplementary Information. [file 41598_2022_25467_MOESM1_ESM.docx]

**Supplemental Appendix**

**Differential diagnosis of common etiologies of left ventricular hypertrophy using a hybrid CNN-LSTM model**

*Hwang I-C, Choi D, et al*. Deep learning for LVH differential diagnosis

1. **Detailed Methods Page 2**
2. **Supplementary Figure Page 5**
3. **Supplementary Tables Page 6**
4. **References Page 16**

**I. Detailed Methods**

**Echocardiography**

Echocardiography equipment used were GE Vivid E9 (transducer M5Sc), GE Vivid E95 (transducer M5Sc), Philips Epic7 (transducer S5-1 and X5-1), Philips Epic7D (transducer S5-1 and X5-1), and Philips Epic CVX (transducer S5-1 and X5-1) systems.

**Development of the CNN-LSTM network**

The first step of the deep learning model development for the differential diagnosis of left ventricular hypertrophy (LVH) was the development of a convolutional neural network (CNN)-long short-term memory (LSTM) network, which comprised three components; 12 identical CNNs, a bi-directional convolutional LSTM (bi-directional ConvLSTM) layer, and a multi-label classification block.(1,2) For the 12 identical CNNs, the same CNN was applied to each of the 12 images extracted as a result of image processing. The CNN comprised 5 convolutional blocks, and each convolutional block comprised 2 convolutional layers and $2\times2$ max pooling. The convolutional layer was composed of a $3\times3$ convolutional operation, Rectified Linear Unit (ReLU), and batch normalization, in this order. As the 5 convolutional blocks were passed, the number of feature maps increased sequentially to 64, 96, 128, 192, and 256. Subsequently, a bi-directional ConvLSTM layer reflecting the temporal and spatial connectivity between images was applied. Finally, a multi-label classification block consisting of global average pooling, a fully connected layer, and a sigmoid activation function was applied to predict hypertensive heart disease (HHD), hypertrophic cardiomyopathy (HCM), and light-chain cardiac amyloidosis (ALCA) independently. This step was applied to each of the 5 standard views. Thus, five independent CNN-LSTM networks were trained.

**Aggregate neural network**

The second step of the deep learning model development for the differential diagnosis of LVH was a neural network which aggregated the results obtained in the first step. The outputs obtained from the global average pooling of the multi-label classification block in each of 5 independent CNN-LSTM networks were concatenated to compose the input. Thus, the input size was $256\times5=1,280$ features. The neural network comprised two fully connected layers and a sigmoid activation function for the individual classification of 3 etiologies of LVH (HHD, HCM, and ALCA). The number of features in the fully connected layer was 1,280 in both layers, and the 3 diseases were independently predicted as the final output.

Binary cross entropy was used as an objective function to train the first and second steps, and the *He*-initialization was used to initialize the weights.(3) The same binary cross entropy was applied to predict HHD, HCM, and ALCA, and the 3 binary cross entropy were simultaneously minimized using the RMSprop optimizer.(4) In the first step, the initial learning rate was set to $5\times{10}^{-5}$, and decayed every 400 steps at a rate 0.94. When the CNN-LSTM network was trained up to approximately 50 epochs for each for the 5 views, each area under the receiver operating characteristic curve (AUC) for HHD, HCM, and ALCA reached almost 1 as each cross entropy converged close to 0. We selected the weight of the epoch with the highest mean of AUCs for HHD, HCM, and ALCA in the validation set as the best model. The batch size was set to 2 considering the memory of the graphic processing unit. In the second step, the process of setting the learning rate and selecting the best model was the same as in the first step. Since the second step does not require the memory of the graphic processing unit as much as in the first step and the number of parameters is small, the batch size was increased to 20 and the number of epochs was 10.

**Class activation mapping**

The region to which the deep learning algorithm reacted sensitively in images was detected using class activation mapping.(5) Class activation mapping was calculated for each LVH etiology (HHD, HCM, and ALCA) by multiplying the layer that passed the first component in step 1 by the weight of the fully connected layer in the multi-label classification block. The regions that reacted sensitively on each view were investigated by class activation mapping in 5 standard views. In addition, to provide objective evidence regarding the deep learning model’s relevance, the highlighted regions on the class activation maps were determined by the expert cardiologist (I-C Hwang) and are summarized in **Supplementary Table S6**.

**II. Supplementary Figure**

**Supplementary Figure S1. ROC curve analysis of the deep learning algorithm for the 5 standard echocardiographic views**

**
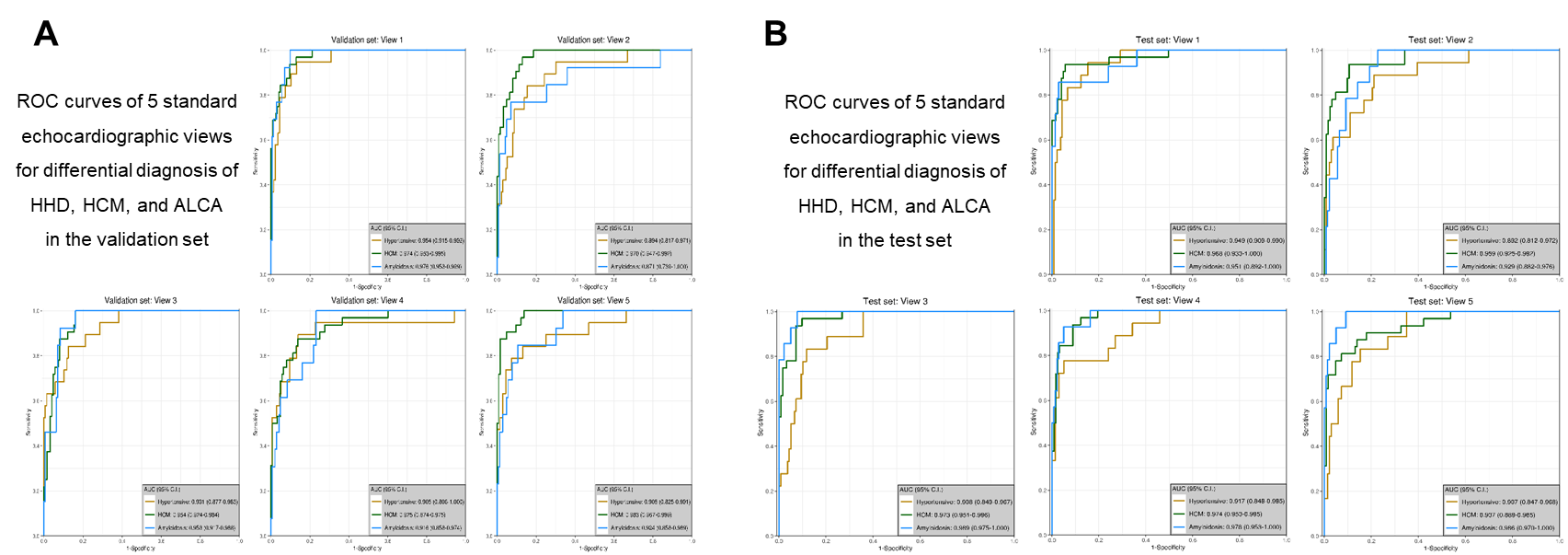
**

The diagnostic accuracy of the deep learning algorithm, as independently developed from 5 standard echocardiographic views, was calculated using the AUC for the validation **(A)**, and test sets **(B)**. This figure was generated using R software v.4.1.1, R Core Team (2021). R: A language and environment for statistical computing. R Foundation for Statistical Computing, Vienna, Austria. URL http:// www.R- project. org/.

Abbreviations: AUC, area under the ROC curve; ALCA, light-chain cardiac amyloidosis; CI, confidence interval; HCM, hypertrophic cardiomyopathy; HHD, hypertensive heart disease; LVH, left ventricular hypertrophy; ROC, receiver operating characteristics curve.

**III. Supplementary Tables**

**Supplementary Table S1. Comparison of the diagnostic accuracy between the CNN-LSTM model and the CNN without LSTM network**

| **Validation set** | **AUC for HHD** | |  | **AUC for HCM** | | |  | **AUC for ALCA** | |
| --- | --- | --- | --- | --- | --- | --- | --- | --- | --- |
|  | **CNN without LSTM** | **CNN-LSTM** |  | **CNN without LSTM** | | **CNN-LSTM** |  | **CNN without LSTM** | **CNN-LSTM** |
| **PLAX** | 0.950 | 0.954 |  | 0.956 | | 0.974 |  | 0.957 | 0.976 |
| **PSAX** | 0.853 | 0.894 |  | 0.931 | | 0.970 |  | 0.879 | 0.871 |
| **A4C** | 0.880 | 0.931 |  | 0.948 | | 0.954 |  | 0.899 | 0.953 |
| **A2C** | 0.831 | 0.905 |  | 0.894 | | 0.925 |  | 0.829 | 0.916 |
| **A3C** | 0.878 | 0.908 |  | 0.942 | | 0.983 |  | 0.928 | 0.924 |
| **Test set** | **AUC for HHD** | | |  | **AUC for HCM** | |  | **AUC for ALCA** | |
|  | **CNN without LSTM** | **CNN-LSTM** |  | **CNN without LSTM** | | **CNN-LSTM** |  | **CNN without LSTM** | **CNN-LSTM** |
| **PLAX** | 0.931 | 0.949 |  | 0.945 | | 0.968 |  | 0.960 | 0.951 |
| **PSAX** | 0.861 | 0.892 |  | 0.934 | | 0.959 |  | 0.854 | 0.929 |
| **A4C** | 0.880 | 0.908 |  | 0.942 | | 0.973 |  | 0.961 | 0.989 |
| **A2C** | 0.787 | 0.917 |  | 0.906 | | 0.974 |  | 0.889 | 0.978 |
| **A3C** | 0.859 | 0.907 |  | 0.921 | | 0.937 |  | 0.959 | 0.986 |

The diagnostic performance of the CNN-LSTM algorithm was compared with that of the CNN algorithm without LSTM network.

Abbreviations: A2C, apical 2-chamber view; A3C, apical 3-chamber view; A4C, apical 4-chamber view; ALCA, light-chain cardiac amyloidosis; CNN-LSTM, convolutional neural network-long short-term memory; HCM, hypertrophic cardiomyopathy; HHD, hypertensive heart disease; LVH, left ventricular hypertrophy; PLAX, parasternal long-axis view; PSAX, parasternal short-axis view.

**Supplementary Table S2. Diagnostic accuracy of the CNN-LSTM model for LVH etiology**

| **Echocardiographic**  **view** | **HHD** | | | | |  | **HCM** | | | | |  | **ALCA** | | | | |
| --- | --- | --- | --- | --- | --- | --- | --- | --- | --- | --- | --- | --- | --- | --- | --- | --- | --- |
|  | **Echo expert** | | **CNN-LSTM model** | | |  | **Echo expert** | | **CNN-LSTM model** | | |  | **Echo expert** | | **CNN-LSTM model** | | |
| **PLAX** | **Value** | **Count** | **Value** | **Count** | **95% CI** |  | **Value** | **Count** | **Value** | **Count** | **95% CI** |  | **Value** | **Count** | **Value** | **Count** | **95% CI** |
| Sensitivity | 38.9% | 7/18 | 83.3% | 15/18 | 58.6–96.4% |  | 75.0% | 24/32 | 93.8% | 30/32 | 79.2–99.2% |  | 64.3% | 9/14 | 85.7% | 12/14 | 57.2–98.2% |
| Specificity | 85.4% | 117/137 | 88.3% | 121/137 | 81.7–93.2% |  | 93.5% | 115/123 | 85.4% | 105/123 | 77.9–91.1% |  | 94.3% | 133/141 | 87.9% | 124/141 | 81.4–92.8% |
| PPV | 25.9% | 7/27 | 48.4% | 15/31 | 30.2–66.9% |  | 75.0% | 24/32 | 62.5% | 30/48 | 47.4–76.0% |  | 52.9% | 9/17 | 41.4% | 12/29 | 23.5–61.1% |
| NPV | 91.4% | 117/128 | 97.6% | 121/124 | 93.1–99.5% |  | 93.5% | 115/123 | 98.1% | 105/107 | 93.4–99.8% |  | 96.4% | 133/138 | 98.4% | 124/126 | 94.4–99.8% |
| **PSAX** |  |  | **Value** | **Count** | **95% CI** |  |  |  | **Value** | **Count** | **95% CI** |  |  |  | **Value** | **Count** | **95% CI** |
| Sensitivity | 50.0% | 9/18 | 77.8% | 14/18 | 52.4–93.6% |  | 75.0% | 24/32 | 93.8% | 30/32 | 79.2–99.2% |  | 14.3% | 2/14 | 64.3% | 9/14 | 35.1–87.2% |
| Specificity | 85.4% | 117/137 | 82.5% | 113/137 | 75.1–88.4% |  | 89.4% | 110/123 | 86.2% | 106/123 | 78.8–91.7% |  | 97.9% | 138/141 | 90.8% | 128/141 | 84.7–95.0% |
| PPV | 31.0% | 9/29 | 36.8% | 14/38 | 21.8–54.0% |  | 64.9% | 24/37 | 63.8% | 30/47 | 48.5–77.3% |  | 40.0% | 2/5 | 40.9% | 9/22 | 20.7–63.6% |
| NPV | 92.9% | 117/126 | 96.6% | 113/117 | 91.5–99.1% |  | 93.2% | 110/118 | 98.1% | 106/108 | 93.5–99.8% |  | 92.0% | 138/150 | 96.2% | 128/133 | 91.4–98.8% |
| **A4C** |  |  | **Value** | **Count** | **95% CI** |  |  |  | **Value** | **Count** | **95% CI** |  |  |  | **Value** | **Count** | **95% CI** |
| Sensitivity | 55.6% | 10/18 | 77.8% | 14/18 | 52.4–93.6% |  | 75.0% | 24/32 | 96.9% | 31/32 | 83.8–99.9% |  | 28.6% | 4/14 | 100.0% | 14/14 | 76.8–100.0% |
| Specificity | 89.1% | 122/137 | 89.8% | 123/137 | 83.4–94.3% |  | 91.9% | 113/123 | 77.2% | 95/123 | 68.8–84.3% |  | 96.5% | 136/141 | 87.9% | 124/141 | 81.4–92.8% |
| PPV | 40.0% | 10/25 | 50.0% | 14/28 | 30.6–69.4% |  | 70.6% | 24/34 | 52.5% | 31/59 | 39.1–65.7% |  | 44.4% | 4/9 | 45.2% | 14/31 | 27.3–64.0% |
| NPV | 93.8% | 122/130 | 96.9% | 123/127 | 92.1–99.1% |  | 93.4% | 113/121 | 99.0% | 95/96 | 94.3–100.0% |  | 93.2% | 136/146 | 100.0% | 124/124 | 97.1–100.0% |
| **A2C** |  |  | **Value** | **Count** | **95% CI** |  |  |  | **Value** | **Count** | **95% CI** |  |  |  | **Value** | **Count** | **95% CI** |
| Sensitivity | 50.0% | 9/18 | 77.8% | 14/18 | 52.4–93.6% |  | 78.1% | 25/32 | 96.9% | 31/32 | 83.8–99.9% |  | 35.7% | 5/14 | 100.0% | 14/14 | 76.8–100.0% |
| Specificity | 86.1% | 118/137 | 86.1% | 118/137 | 79.2–91.4% |  | 91.1% | 112/123 | 87.8% | 108/123 | 80.7–93.0% |  | 97.2% | 137/141 | 73.8% | 104/141 | 65.7–80.8% |
| PPV | 32.1% | 9/28 | 42.4% | 14/33 | 25.5–60.8% |  | 69.4% | 25/36 | 67.4% | 31/46 | 52.0–80.5% |  | 55.6% | 5/9 | 27.5% | 14/51 | 15.9–41.7% |
| NPV | 92.9% | 118/127 | 96.7% | 118/122 | 91.8–99.1% |  | 94.1% | 112/119 | 99.1% | 108/109 | 95.0–100.0% |  | 93.8% | 137/146 | 100.0% | 104/104 | 96.5–100.0% |
| **A3C** |  |  | **Value** | **Count** | **95% CI** |  |  |  | **Value** | **Count** | **95% CI** |  |  |  | **Value** | **Count** | **95% CI** |
| Sensitivity | 44.4% | 8/18 | 66.7% | 12/18 | 41.0–86.7% |  | 75.0% | 24/32 | 90.6% | 29/32 | 75.0–98.0% |  | 42.9% | 6/14 | 85.7% | 12/14 | 57.2–98.2% |
| Specificity | 92.0% | 126/137 | 89.1% | 122/137 | 82.6–93.7% |  | 94.3% | 116/123 | 77.2% | 95/123 | 68.8–84.3% |  | 95.7% | 135/141 | 95.7% | 135/141 | 91.0–98.4% |
| PPV | 42.1% | 8/19 | 44.4% | 12/27 | 25.5–64.7% |  | 77.4% | 24/31 | 50.9% | 29/57 | 37.3–64.4% |  | 50.0% | 6/12 | 66.7% | 12/18 | 41.0–86.7% |
| NPV | 92.6% | 126/136 | 95.3% | 122/128 | 90.1–98.3% |  | 93.5% | 116/124 | 96.9% | 95/98 | 91.3–99.4% |  | 94.4% | 135/143 | 98.5% | 135/137 | 94.8–99.8% |

The diagnostic accuracy for the differential diagnosis of LVH (HHD, HCM, and ALCA) was assessed using the sensitivity, specificity, positive predictive value (PPV), and negative predictive value (NPV), for each standard echocardiographic view.

Abbreviations: A2C, apical 2-chamber view; A3C, apical 3-chamber view; A4C, apical 4-chamber view; ALCA, light-chain cardiac amyloidosis; HCM, hypertrophic cardiomyopathy; HHD, hypertensive heart disease; PLAX, parasternal long-axis view; PSAX, parasternal short-axis view.

**Supplementary Table S3. Comparison of diagnostic performance for LVH etiology between echocardiography experts and the aggregate network**

|  | **HHD** | | | | |  | **HCM** | | | | |  | **ALCA** | | | | |
| --- | --- | --- | --- | --- | --- | --- | --- | --- | --- | --- | --- | --- | --- | --- | --- | --- | --- |
|  | **Echo expert** | | **Aggregate network** | | |  | **Echo expert** | | **Aggregate network** | | |  | **Echo expert** | | **Aggregate network** | | |
|  | **Value** | **Count** | **Value** | **Count** | **95% CI** |  | **Value** | **Count** | **Value** | **Count** | **95% CI** |  | **Value** | **Count** | **Value** | **Count** | **95% CI** |
| **Sensitivity** | 52.8% | 19/36 | 72.2% | 13/18 | 46.5% - 90.3% |  | 85.9% | 55/64 | 90.6% | 29/32 | 75.0% - 98.0% |  | 50.0% | 14/28 | 92.9% | 13/14 | 66.1% - 99.8% |
| **Specificity** | 89.1% | 244/274 | 94.2% | 129/137 | 88.8% - 97.4% |  | 93.1% | 229/246 | 93.5% | 115/123 | 87.6% - 97.2% |  | 96.1% | 271/282 | 95.7% | 135/141 | 91.0% - 98.4% |
| **PPV** | 38.8% | 19/49 | 61.9% | 13/21 | 38.4% - 81.9% |  | 76.4% | 55/72 | 78.4% | 29/37 | 61.8% - 90.2% |  | 56.0% | 14/25 | 68.4% | 13/19 | 43.4% - 87.4% |
| **NPV** | 93.5% | 244/261 | 96.3% | 129/134 | 91.5% - 98.8% |  | 96.2% | 229/238 | 97.5% | 115/118 | 92.7% - 99.5% |  | 95.1% | 271/285 | 99.3% | 135/136 | 96.0% - 100.0% |

The diagnostic performance of the aggregate network of the CNN-LSTM algorithm was assessed using the sensitivity, specificity, positive predictive value (PPV), and negative predictive value (NPV), for each etiology of LVH.

Abbreviations: A2C, apical 2-chamber view; A3C, apical 3-chamber view; A4C, apical 4-chamber view; ALCA, light-chain cardiac amyloidosis; CNN-LSTM, convolutional neural network-long short-term memory; HCM, hypertrophic cardiomyopathy; HHD, hypertensive heart disease; LVH, left ventricular hypertrophy; PLAX, parasternal long-axis view; PSAX, parasternal short-axis view.

**Supplementary Table S4. Comparison of the AUCs according to the number of extracted images per cardiac cycle**

| **Hyperparameter**  **(number of images extracted**  **per 1 cardiac cycle)** | **Echocardiographic views** | **AUCs** | | |
| --- | --- | --- | --- | --- |
|  |  | **HHD** | **HCM** | **ALCA** |
| **4 images/cardiac cycle** | **PLAX** | 0.950 | 0.968 | 0.963 |
|  | **PSAX** | 0.889 | 0.959 | 0.932 |
|  | **A4C** | 0.927 | 0.960 | 0.995 |
|  | **A2C** | 0.873 | 0.972 | 0.968 |
|  | **A3C** | 0.910 | 0.954 | 0.980 |
|  | **Aggregate** | 0.959 | 0.986 | 0.998 |
| **8 images/cardiac cycle** | **PLAX** | 0.946 | 0.961 | 0.965 |
|  | **PSAX** | 0.905 | 0.957 | 0.922 |
|  | **A4C** | 0.891 | 0.976 | 0.993 |
|  | **A2C** | 0.905 | 0.964 | 0.969 |
|  | **A3C** | 0.890 | 0.945 | 0.980 |
|  | **Aggregate** | 0.961 | 0.985 | 0.995 |
| **12 images/cardiac cycle** | **PLAX** | 0.949 | 0.968 | 0.951 |
|  | **PSAX** | 0.892 | 0.959 | 0.929 |
|  | **A4C** | 0.908 | 0.973 | 0.989 |
|  | **A2C** | 0.917 | 0.974 | 0.978 |
|  | **A3C** | 0.907 | 0.937 | 0.986 |
|  | **Aggregate** | 0.962 | 0.982 | 0.996 |
| **16 images/cardiac cycle** | **PLAX** | 0.951 | 0.972 | 0.952 |
|  | **PSAX** | 0.824 | 0.942 | 0.929 |
|  | **A4C** | 0.923 | 0.973 | 0.988 |
|  | **A2C** | 0.900 | 0.953 | 0.969 |
|  | **A3C** | 0.860 | 0.940 | 0.976 |
|  | **Aggregate** | 0.960 | 0.980 | 0.992 |

The AUCs of the CNN-LSTM algorithm for each echocardiographic view (PLAX, PSAX, A4C, A2C, and A3C) and the aggregate network were compared according to the number of images extracted per 1 cardiac cycle, for each etiology of LVH.

Abbreviations: AUC, area under the receiver operating characteristic curve; A2C, apical 2-chamber view; A3C, apical 3-chamber view; A4C, apical 4-chamber view; ALCA, light-chain cardiac amyloidosis; CNN-LSTM, convolutional neural network-long short-term memory; HCM, hypertrophic cardiomyopathy; HHD, hypertensive heart disease; LVH, left ventricular hypertrophy; PLAX, parasternal long-axis view; PSAX, parasternal short-axis view.

**Supplementary Table S5. Comparison of the AUCs between the 2D CNN-LSTM with aggregate network and the 3D CNN model**

| **Type of algorithm** | **Echocardiographic views** | **AUCs** | | |
| --- | --- | --- | --- | --- |
|  |  | **HHD** | **HCM** | **ALCA** |
| **2D CNN-LSTM**  **(12 images/cardiac cycle)** | **PLAX** | 0.949 | 0.968 | 0.951 |
|  | **PSAX** | 0.892 | 0.959 | 0.929 |
|  | **A4C** | 0.908 | 0.973 | 0.989 |
|  | **A2C** | 0.917 | 0.974 | 0.978 |
|  | **A3C** | 0.907 | 0.937 | 0.986 |
|  | **Aggregate** | 0.962 | 0.982 | 0.996 |
| **3D CNN**  **(12 images/cardiac cycle)** | **PLAX** | 0.949 | 0.967 | 0.968 |
|  | **PSAX** | 0.868 | 0.942 | 0.784 |
|  | **A4C** | 0.886 | 0.967 | 0.964 |
|  | **A2C** | 0.925 | 0.931 | 0.943 |
|  | **A3C** | 0.941 | 0.962 | 0.944 |
|  | **Aggregate** | 0.966 | 0.987 | 0.980 |
| **3D CNN**  **(16 images/cardiac cycle)** | **PLAX** | 0.965 | 0.960 | 0.977 |
|  | **PSAX** | 0.880 | 0.963 | 0.854 |
|  | **A4C** | 0.945 | 0.974 | 0.961 |
|  | **A2C** | 0.897 | 0.949 | 0.975 |
|  | **A3C** | 0.935 | 0.962 | 0.953 |
|  | **Aggregate** | 0.972 | 0.986 | 0.987 |

The AUCs of the 2D CNN-LSTM algorithm with aggregate network were compared with the 3D CNN algorithm. The number of echocardiographic images per 1 cardiac cycle was tested for 12 images/cardiac cycle and 16 images/cardiac cycle for the 3D CNN algorithm.

Abbreviations: 2D, 2-dimensional; 3D, 3-dimensional; AUC, area under the receiver operating characteristic curve; A2C, apical 2-chamber view; A3C, apical 3-chamber view; A4C, apical 4-chamber view; ALCA, light-chain cardiac amyloidosis; CNN-LSTM, convolutional neural network-long short-term memory; HCM, hypertrophic cardiomyopathy; HHD, hypertensive heart disease; LVH, left ventricular hypertrophy; PLAX, parasternal long-axis view; PSAX, parasternal short-axis view.

**Supplementary Table S6. Frequencies of highlighted regions on class activation mapping of the test set**

| **Echocardiographic**  **view** | **HHD (n = 18)** | |  | **HCM (n = 32)** | |  | **ALCA (n = 14)** | |
| --- | --- | --- | --- | --- | --- | --- | --- | --- |
|  | **Region of interest** | **Frequency** |  | **Region of interest** | **Frequency** |  | **Region of interest** | **Frequency** |
| **PLAX** | Ascending aorta | 77.8% (14/18) |  | LV basal anteroseptum | 96.9% (31/32) |  | Left atrial wall | 78.6% (11/14) |
|  | LV basal inferolateral segment  (with posterior MV leaflet) | 55.6% (10/18) |  | LV basal inferolateral segment | 56.3% (18/32) |  | Pericardium (LV posterior) | 71.4% (10/14) |
|  | Left atrial wall | 55.6% (10/18) |  | LV mid anteroseptum | 37.5% (12/32) |  | RV free wall | 35.7% (5/14) |
| **PSAX** | LV mid anteroseptum (RV insertion site) | 88.9% (16/18) |  | LV mid anteroseptum | 84.4% (27/32) |  | Pericardium (LV posterior) | 92.9% (13/14) |
|  | LV mid inferior segment (RV insertion site) | 66.7% (12/18) |  | LV mid inferior wall | 84.4% (27/32) |  | LV mid anteroseptum | 64.3% (9/14) |
|  | LV mid anterior segment | 55.6% (10/18) |  | Papillary muscles | 78.1% (25/32) |  | Papillary muscles | 64.3% (9/14) |
| **A4C** | RV free wall (apical segment) | 83.3% (15/18) |  | LV mid inferoseptum | 84.4% (27/32) |  | LV basal inferoseptum | 71.4% (10/14) |
|  | LV mid inferoseptum | 61.1% (11/18) |  | LV basal inferoseptum | 75.0% (24/32) |  | LV mid inferoseptum | 57.1% (8/14) |
|  | LV basal inferoseptum | 55.6% (10/18) |  | Papillary muscles | 53.1% (17/32) |  | Left atrial wall | 50.0% (7/14) |
| **A2C** | LV basal inferior segment | 88.9% (16/18) |  | LV basal inferior segment | 87.5% (28/32) |  | LV basal inferior segment | 78.6% (11/14) |
|  | LV mid inferior segment | 83.3% (15/18) |  | LV mid inferior segment | 71.9% (23/32) |  | Left atrial wall | 42.9% (6/14) |
|  | LV apical inferior segment | 27.8% (5/18) |  | LV apical inferior segment | 62.5% (20/32) |  | LV mid inferior segment | 28.6% (4/14) |
| **A3C** | LV basal inferolateral segment | 94.4% (17/18) |  | LV basal inferolateral segment | 68.8% (22/32) |  | LV basal inferolateral segment | 92.9% (13/14) |
|  | LV mid inferolateral segment | 33.3% (6/18) |  | LV apical septum | 59.4% (19/32) |  | Mitral valve | 64.3% (9/14) |
|  | LV mid anteroseptum | 16.7% (3/18) |  | LV mid anteroseptum | 50.0% (16/32) |  | Left atrial wall | 50.0% (7/14) |

Abbreviations: A2C, apical 2-chamber view; A3C, apical 3-chamber view; A4C, apical 4-chamber view; ALCA, light-chain cardiac amyloidosis; CNN-LSTM, convolutional neural network-long short-term memory; HCM, hypertrophic cardiomyopathy; HHD, hypertensive heart disease; LV, left ventricular; MV, mitral valve; PLAX, parasternal long-axis view; PSAX, parasternal short-axis view; RV, right ventricular.

**References**

1. Krizhevsky A, Sutskever I, Hinton GEJAinips. Imagenet classification with deep convolutional neural networks. 2012;25:1097-1105.

2. Xingjian S, Chen Z, Wang H, Yeung D-Y, Wong W-K, Woo W-c. Convolutional LSTM network: A machine learning approach for precipitation nowcasting. Advances in neural information processing systems, 2015:802-810.

3. He K, Zhang X, Ren S, Sun J. Delving deep into rectifiers: Surpassing human-level performance on imagenet classification. Proceedings of the IEEE international conference on computer vision, 2015:1026-1034.

4. Hinton G, Srivastava N, Swersky KJCo. Neural networks for machine learning lecture 6a overview of mini-batch gradient descent. 2012;14:2.

5. Zhou B, Khosla A, Lapedriza A, Oliva A, Torralba A. Learning deep features for discriminative localization. Proceedings of the IEEE conference on computer vision and pattern recognition, 2016:2921-2929.
